# Photothermal Coating on Zinc Alloy for Controlled Biodegradation and Improved Osseointegration

**DOI:** 10.1002/advs.202409051

**Published:** 2025-01-14

**Authors:** Yuchien Hsu, Yunjiao He, Xiao Zhao, Feilong Wang, Fan Yang, Yufeng Zheng, Yongsheng Zhou, Dandan Xia, Yunsong Liu

**Affiliations:** ^1^ Department of Prosthodontics Peking University School and Hospital of Stomatology No.22, Zhongguancun South Avenue, Haidian District Beijing 100081 China; ^2^ School of Materials Science and Engineering Peking University No.5 Yi‐He‐Yuan Road, HaiDian District Beijing 100871 China; ^3^ National Center for Stomatology & National Clinical Research Center for Oral Diseases & National Engineering Research Center of Oral Biomaterials and Digital Medical Devices & Beijing Key Laboratory of Digital Stomatology & NHC Key Laboratory of Digital Stomatology & NMPA Key Laboratory for Dental Materials Peking University School and Hospital of Stomatology No.22, Zhongguancun South Avenue, Haidian District Beijing 100081 China; ^4^ Department of Dental Materials Peking University School and Hospital of Stomatology No.22, Zhongguancun South Avenue, Haidian District Beijing 100081 China

**Keywords:** biodegradation, near‐infrared light, orthopedic implantation, osseointegration, Zn‐Li alloy

## Abstract

Zinc (Zn) and its alloys are promising biomaterials for orthopedic applications due to their degradability and mechanical properties. Zn^2+^ plays a crucial role in bone formation, but excessive early release may cause cytotoxicity and inhibit osseointegration. To solve this, we developed a near‐infrared (NIR) light‐controlled polycaprolactone/copper‐sulfur (PCL/CuS) coating that slows degradation and enhances osseointegration of Zn alloys. The zinc–lithium (Zn–Li) substrate is encapsulated with PCL, reducing Zn^2+^ release and maintaing biocompatibility. Controlled Zn^2+^ release and mild photothermal therapy via CuS nanoparticles promoted osteogenesis. In vitro studies demonstrated enhanced cell proliferation and osteogenic differentiation. In vivo Micro‐Computed Tomography (Micro‐CT), Scanning Electron Microscopy‐Energy Dispersive Spectroscopy (SEM‐EDS), and immunohistochemical analyses confirmed improved osseointegration. Mechanistic studies using RNA sequencing and Western blotting revealed that the coating promotes osteogenesis by activating the Wnt/β‐catenin and inhibiting NF‐κB pathways. This NIR light‐controlled PCL/CuS coating successfully regulates Zn alloy degradation, enhances osseointegration via controlled Zn^2+^ release and mild photothermal therapy effct, presenting a promising avenue for orthopedic biomaterials.

## Graphical abstract

An NIR‐responsive PCL/CuS coating on Zn–Li alloy were designed to promote osseointegration. PCL encapsulation regulates Zn^2^⁺ release, while CuS nanoparticles enable mild NIR‐driven photothermal therapy. This synergy of controlled Zn^2^⁺ release and mild photothermal therapy effectively enhances bone integration, showing strong potential in orthopedic application.

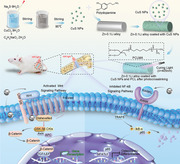

## 1 Introduction

Zinc (Zn) and its alloys have become promising biomaterials in orthopedic applications in recent years. Compared to titanium, bioceramics, polymers, and other bone substitutes, Zn alloys offer key advantages including their degradability, bioactive ion release, and mechanical compatibility with bone tissue.^[^ ^1^ ^]^ Zn is the second most prevalent metal in the human body, crucial for the formation, development, mineralization, and maintenance of the human skeleton.^[^ ^2^ ^]^ An initial study indicated that zinc–lithium (Zn–Li) alloys demonstrated higher tensile strength and elongation compared to pure Zn due to grain refinement.^[^ ^3^ ^]^ Apart from this, excessive degradation rates of Zn alloys at the early stage may lead to excessive Zn^2+^ release, causing cytotoxicity and inhibiting osseointegration. Surface modification is an effective method for altering degradation behavior. There is an urgent need to find a suitable surface modification for Zn alloys that will control the degradation rate and enhance osseointegration.

Lately, most surface modifications of Zn alloys have aimed at providing a protective effect to reduce the degradation rate and enhance biocompatibility. Phosphate conversion coating,^[^ ^4^ ^]^ biomimetic deposition,^[^ ^5^ ^]^ organic and polymer coating,^[^ ^6^ ^]^ anodic oxidation,^[^ ^7^ ^]^ and atomic layer deposition^[^ ^3b^ ^]^ have been employed to provide protective films, significantly improving corrosion resistance. Recently, Chen et al. proposed a coating that combines CO₂ plasma treatment with choline phosphate chitosan grafting on a Zinc‐1Magnesium (Zn1Mg) alloy surface to enhance biocompatibility, reduce ion release, and create an immune‐supportive environment for effective bone repair.^[^ ^8^ ^]^ Additionally, some researchers have combined drugs with coatings to boost implant efficacy. An aliphatic polycarbonate coating on Zn–Li alloys with simvastatin achieves a controlled release of Zn^2^⁺ and simvastatin, promoting osteogenesis, angiogenesis, and antibacterial effects.^[^ ^6b^ ^]^ However, the methods for effectively controlling the degradation rate of Zn alloys to improve the osseointegration by combining external stimulation is still limited. Clearly, a suitable surface that can incorporate external stimulation for dual effects to address the above‐mentioned issues is in demand.

Polycaprolactone (PCL) is a semicrystalline aliphatic polyester consisting of repeating units comprising five methylene groups and one ester group. As a biodegradable polyester, PCL offers several advantages for use as a biomaterial coating. Its high biocompatibility ensures minimal immune response and toxicity when implanted in the body, making it suitable for various medical devices and tissue engineering applications.^[^ ^9^ ^]^ On the surface of ZM21 Mg alloy, Singh et al.^[^ ^10^ ^]^ compared the corrosion resistance of TiO_2_‐Hydroxyapatite (HA) composite coating and TiO_2_‐HA‐PCL coating. They discovered that TiO_2_‐HA‐PCL coating significantly increased the corrosion resistance of Mg alloy, and the bonding strength of the coating was minimally impacted in the immersion experiments. Zhao et al.^[^ ^11^ ^]^ developed a dual pH/NIR‐responsive and self‐healing PCL‐diacrylate/polydopamine (PCL‐DA/PDA) bilayer coating on Mg alloys. By adding drug‐loaded nanocapsules to the PCL‐DA/PDA coating, it was possible to kill bacteria, promote osteogenesis, and angiogenesis while also effectively shielding the Mg substrate from corrosion for up to 240 h in Hank's balanced salt solution. Therefore, we propose that the PCL coatings on Zn–Li alloys effectively slow down the degradation rate of Zn alloys and increase biocompatibility.

Near‐infrared (NIR) light‐assisted photothermal therapy (PTT) involves the absorption of NIR light by specific materials, leading to localized heating of the implant site. PTT stands out against conventional treatments by offering greater precision, non‐invasive application, and finely‐tuned spatial and temporal selectivity.^[^ ^12^ ^]^ Notably, mild photothermal therapy at 40–42 °C triggered with NIR light promotes osseointegration.^[^ ^13^ ^]^ To achieve high efficacy, a photothermal agent (PTA) with high photothermal conversion efficiency is required for PTT.^[^ ^14^ ^]^ Various forms of PTA, particularly nanoparticles (NPs) exhibiting significant absorbance in the NIR region, have been studied. Copper–sulfur (CuS) NPs are of interest due to their low cost, minimal cytotoxicity, and remarkable NIR optical absorption, and high molar extinction coefficient.^[^ ^15^ ^]^ In addition to acting as a PTA, Cu induces the proliferation of human endothelial cells and stimulates the secretion of Vascular Endothelial Growth Factor (VEGF) in vitro, thereby initiating angiogenesis.^[^ ^16^ ^]^ Apart from its essential role in vascularization, Cu contributes to the growth and viability of osteoblastic cells.^[^ ^17^ ^]^ Considering its multiple functions, a NIR‐controlled coating based on CuS NPs may be a promising approach for orthopedic applications.

**Scheme 1:**
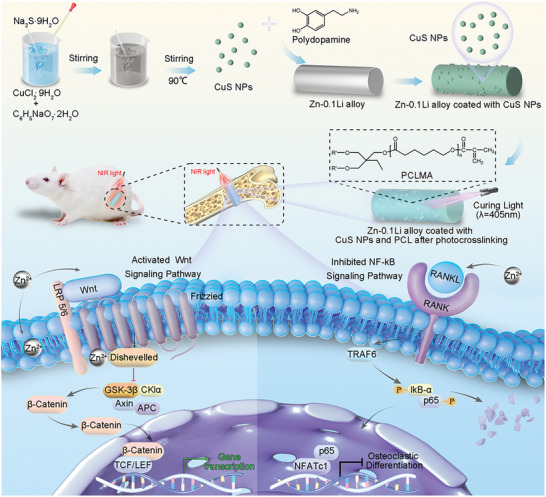
A synthesis method of the NIR light‐controlled PCL/CuS coated Zn–Li alloy and the related mechanisms for the promotion of osseointegration.

In this research, we successfully engineered a PCL/CuS‐coated Zn‐0.1Li alloy with NIR light control. PCL was used to encapsulate the Zn–Li alloy, providing a protective film after photocrosslinking. The CuS NPs were used as PTA to convert light energy into heat, and Cu played a crucial role in promoting angiogenesis and bone formation. Controllable Zn^2+^ concentration combined with mild PTT triggered with NIR light was used to improve osseointegration. This innovative approach simultaneously achieved controllable degradation of Zn‐based BMs and improved osseointegration (**Scheme** **1**). After the coating was prepared, the surface characteristics, degradation performance, photothermal performance, biocompatibility, and bone integration‐promoting performance of the NIR light‐controlled PCL/CuS‐coated Zn‐0.1Li alloy were evaluated in vitro. A Sprague–Dawley (SD) rat femoral bone defect model was used to evaluate the osseointegration effect and degradation behavior in vivo. In addition, RNA sequencing and Western blotting revealed that the NIR light‐controlled PCL/CuS coatings promoted osseointegration by activating the Wnt/β‐catenin and NF‐κB signaling pathways to regulate osteogenic differentiation.

## 2 Results and Discussion

### 2.1 Synthesis and Characterization of PCL/CuS Coatings

**Figure 1:**
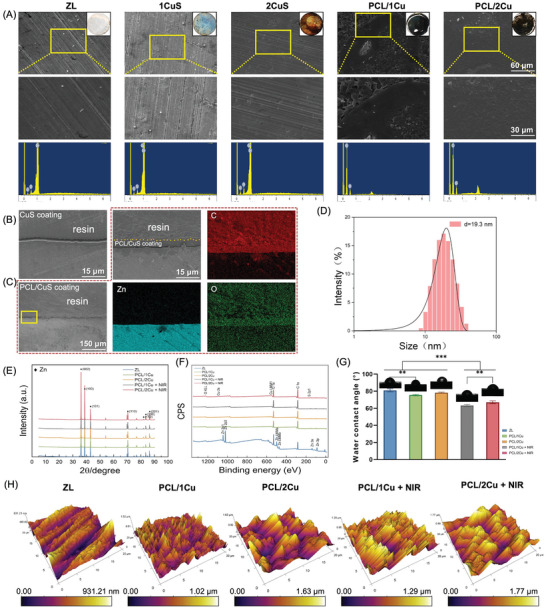
Surface characterization and composition analysis. (A) General appearance (upper right) and surface morphology of ZL, CuS coatings, PCL/1Cu and PCL/2Cu coatings (lower panels are magnified) and their corresponding EDS profiles; (B, C) Cross‐sectional morphologies and EDS map of CuS and PCL/CuS coatings; (D) DLS analysis of CuS NPs; (E) XRD patterns; (F) XPS wide‐scan spectra; (G) Water‐air contact angles and summary results; (H) 3D surface morphology. n = 3 (^**^ *p* < 0.01, ^***^ *p* < 0.001).

**Figure** **1**A shows the morphology of the bare Zn‐0.1Li alloy (referred to ZL as following) substrate with PDA‐treated CuS NPs and PCL coatings light‐cured from Polycaprolactone Methacryloyl (PCLMA). By mixing CuS NPs with PDA solution at a volume ratio of 0.5:1, PCL/CuS coatings of various Cu concentrations (referred to as PCL/1Cu and PCL/2Cu, respectively) were obtained. The color of the coating was visually observed to darken with increasing CuS NPs content. Figure 1A also shows the surface microstructure of the ZL substrate, CuS coatings with varying CuS NPs content, and materials with a thin layer of PCL applied. SEM images revealed that the coatings uniformly covered the material surface. Cross‐sectional SEM images showed that, due to the adhesive effect and chelating functional groups of PDA, the CuS coating was tightly attached to the ZL substrate surface with no visible gaps between them (Figure 1B). In Figure 1C, a slight gap can be observed between the PCL and CuS layers, primarily because no chemical bond forms between the PCL and the CuS coating. PCL underwent light‐curing, converting liquid PCLMA into solid PCL, achieving overall coating integrity through physical encapsulation and thereby providing a protective function. Measured by dynamic light scattering (DLS), the average size of the CuS NPs shown in Figure 1D was 19.3 nm (polydispersity index = 0.071). Energy Dispersive X‐ray Spectroscopy (EDS) confirmed the loading of Cu onto the ZL substrate surface. After the addition of the PCL coating, only C and O were detected, which was consistent with complete encapsulation of the underlying metal.

X‐ray diffraction (XRD) analysis revealed characteristic peaks of Zn in all four groups, with the most notable peak was observed around a 2θ value of ≈36.5°, with a secondary peak observed near 39.5°. However, characteristic peaks of PCL were absent in the samples coated with PCL, including the PCL/1Cu, PCL/2Cu, PCL/1Cu + NIR, and PCL/2Cu + NIR groups (Figure 1E). It is speculated that this may be due to the low content and thin coating of loaded PCL. The characteristic peaks of PCL may have been overshadowed by the peaks of the Zn, or due to the semi‐crystalline structure of PCL itself, making it difficult to observe clear characteristic peaks in the XRD spectra. Particularly in the form of thin films or coatings, the crystalline structure of PCL may be influenced by surface or structural factors.^[^ ^18^ ^]^ To verify the presence of PCL, X‐ray photoelectron spectroscopy (XPS) analysis was conducted. A characteristic peak of PCL was observed in the C 1s peak, with a position at ≈287.5 eV. Additionally, no characteristic peaks of Cu and S were observed, indicating that the PCL coating effectively encapsulated the sample surface (Figure 1F). This result further supports the successful loading of PCL and provides strong evidence for our speculation. Moreover, NIR irradiation did not alter the crystal phase structure of the sample surface, indicating that NIR irradiation does not affect the surface stability of the material.

The water‐air contact angle indicates the hydrophilic or hydrophobic characteristics of a surface. Application of the PCL/CuS coating lowered the contact angle of the PCL/1Cu group compared to the ZL group (*P* < 0.01), decreasing it from 81.00° to 75.43°. Following NIR laser exposure, the PCL coating melted, resulting in a further decrease in the contact angle of the PCL/1Cu group from 75.43° to 63.22°, a reduction of 12.21°, indicating an increase in the material surface hydrophilicity (*P* < 0.001; Figure 1G). Atomic force microscopy (AFM) imaging was used to analyze the surface morphology of the samples. Within the scanning area, scratches generated by polishing were observed in the ZL group. Additionally, height maps obtained by AFM revealed the vertical height distribution of the sample surface. Different height variations were observed within the scanning area. With NIR light irradiation, the highest points of the PCL/1Cu and PCL/2Cu groups decreased by 0.51 and 0.48 µm, respectively (Figure 1H).

Results indicated that in samples coated with PCL, characteristic peaks of Zn were observed instead of PCL's characteristic peaks. This could be attributed to the low content and thinness of the loaded PCL coating. The preparation method and parameters of the PCL coating played a significant role in its performance and functionality. Further research could optimize the preparation process of PCL coatings, adjusting their thickness, structure, and chemical composition to achieve better biocompatibility and protective effects.^[^ ^19^ ^]^ Additionally, exploring the composite application of PCL with different types of functional nanomaterials could further expand its potential applications in the biomedical field.

### 2.2 Degradation Behavior

**Figure 2:**
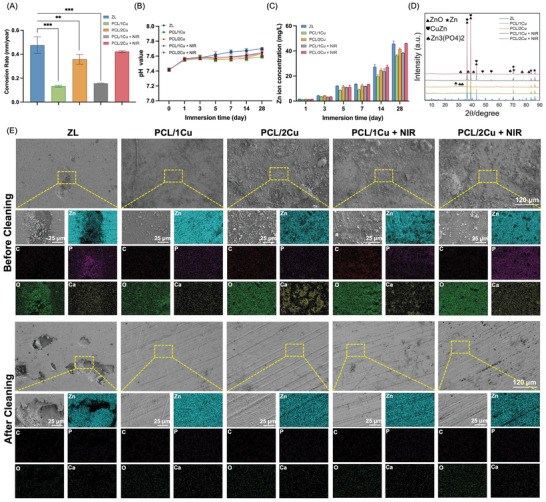
Degradation behavior of samples immersed in SBF. (A) Corrosion rate; (B) pH evolution over immersion time; (C) Concentration of Zn^2+^ after immersing; (D) XRD patterns and obtained from degraded PCL/CuS coated ZL alloys at 7 days; (E) Surface morphology and EDS mapping of different groups after immersing for 7 d. n = 3 (^*^ *p* < 0.05, ^**^ *p* < 0.01, ^***^ *p* < 0.001, when compared with ZL group).

**Figure** **2** presents the degradation behavior of each group for 1, 3, 5, 7, 14, and 28 day‐immersion in simulated body fluid (SBF). Figure 2A shows that the average corrosion rate calculated according to weight loss of the ZL group is 0.475 ± 0.055 µm year^−1^, while for the groups with PCL/CuS coatings (1Cu and 2Cu), the average corrosion rates are 0.131 ± 0.007 µm year^−1^ and 0.357 ± 0.033 µm year^−1^, respectively. NIR light irradiation led to a small increase in the degradation rate, which can better meet the needs of orthopedic degradation, with the rate increasing to 0.157 ± 0.003 µm year^−1^ in the PCL/1Cu + NIR group and 0.422 ± 0.006 µm year^−1^ in the PCL/2Cu + NIR group. This may be due to the increase in temperature, which can influence the diffusion coefficient of ions and the charge transfer rate, thereby impacting the degradation behavior of zinc alloys. Additionally, based on the Arrhenius equation,^[^ ^20^ ^]^ temperature has a well‐established relationship with reaction rates, which helps explain why NIR light exposure can accelerate the degradation process of zinc alloys.^[^ ^21^ ^]^ However, the PCL functions as a barrier, mitigating the effects of elevated temperatures by controlling ion release and reducing direct exposure to the corrosive environment, thus significantly slowing the overall corrosion rate compared to bare ZL alloy. Based on the experimental data, the pH values of the samples in simulated body fluid were observed to vary (Figure 2B). In the ZL group, the pH remained between ≈7.46 ± 0.04 and 7.49 ± 0.05 during the immersion period from 1 to 28 days. With or without NIR light irradiation, the pH values of the groups with PCL/CuS coatings (including PCL/1Cu and PCL/2Cu) were slightly lower than those of the ZL group. In the first 7 days of immersion, Zn^2+^ release decreased significantly from 13.46 ± 0.27 mg L^−1^ in the ZL group to 8.67 ± 0.33 mg L^−1^ in the PCL/1Cu group (*P* < 0.001). After 28 days of immersion, PCL/1Cu and PCL/1Cu + NIR groups showed a decrease in Zn^2+^ release, with the Zn^2+^ concentration reaching 38.26 ± 0.58 mg L^−1^ for the PCL/1Cu + NIR group, compared with 45.84 ± 1.69 mg L^−1^ for the ZL group (Figure 2C). Figure 2D presents the surface microstructure and EDS maps of the samples after 7 days of immersion, including before and after chromic acid cleaning. Chromic acid was employed to remove corrosion products from the surface of the bare Zn alloy, allowing for clearer observation of the Zn alloys beneath the coating. In the PCL/CuS coating groups, initial polishing marks were still visible, with only a small amount of minor degradation pits. In the ZL group, evident corrosion traces in the form of crater‐like pits were observed. Further analysis of the corrosion product composition by EDS revealed that they were mainly consisting of Zn, O, and P. The observed elemental Ca might correspond to the deposition of Ca from the SBF.

The degradation behavior of Zn‐BMs is crucial in understanding their long‐term performance and biocompatibility in physiological environments. In this study, the immersion of ZL alloys in SBF provided insights into their degradation kinetics and the influence of PCL/CuS protective coatings, on corrosion rates. In the physiological environment, Zn alloys primarily degrade through the process of Zn anodic oxidation to form Zn^2^⁺ and simultaneous cathodic reduction of O₂ (Equations (1) and (2)).^[^ ^22^ ^]^ This process elevates local pH and increases the concentration of Zn^2^⁺, promoting the formation of ZnO and Zn(OH)₂ (Equations (3) and (4)), which subsequently creates a protective barrier layer on the material surface.^[^ ^23^ ^]^ The depletion of OH‐ led to a decrease in pH within the system. Here, the pH of the bare Zn alloy increased rapidly during the first 14 days due to the rapid degradation of the Zn alloy substrate,^[^ ^6^, ^24^ ^]^ while the PCL/CuS coated Zn alloy showed a relatively low pH (Figure 2B). And we conducted XRD analysis on the corrosion products and found that ZnO was the primary phase, rather than Zn(OH)₂ (Figure 2D). This result can likely be attributed to several factors: 1) ZnO is the thermodynamically stable phase within the pH range of 7.7–11,^[^ ^25^ ^]^ 2) the physiological temperature of ≈37 °C favors the formation of ZnO over Zn(OH)₂,^[^ ^26^ ^]^ and 3) Zn(OH)₂ tends to dehydrate at 37 °C, converting gradually into ZnO.^[^ ^27^ ^]^ Meanwhile, Zn_3_(PO_4_)_2_ can also be seen in the corrosion products in the XRD analysis results, which is consistent with the EDS results (Figure 2D,E). This is due to the fact that the subsequent generation of hydrated Zn phosphate is thermodynamically more stable in a physiological environment.^[^ ^23^ ^]^

(1) $$\mathrm{Zn}\;\to \;{\mathrm{Zn}}^{2+}+2e^{-}$$

(2) $$2H_{2}O+O_{2}+4e^{-}\to 4OH^{-}$$

(3) $$Zn^{2+}+2OH^{-}⇋\mathrm{Zn}{\left(\mathrm{OH}\right)}_{2}$$

(4) $$\mathrm{Zn}{\left(\mathrm{OH}\right)}_{2}\to \mathrm{ZnO}+H_{2}O$$

(5) $$3{\mathrm{Zn}}^{2+}+2\mathrm{HP}{O_{4}}^{2-}+2{\mathrm{OH}}^{-}+2H_{2}O\to {\mathrm{Zn}}_{3}{\left({\mathrm{PO}}_{4}\right)}_{2}\cdot 4H_{2}O$$

### 2.3 Photothermal Effect

**Figure 3:**
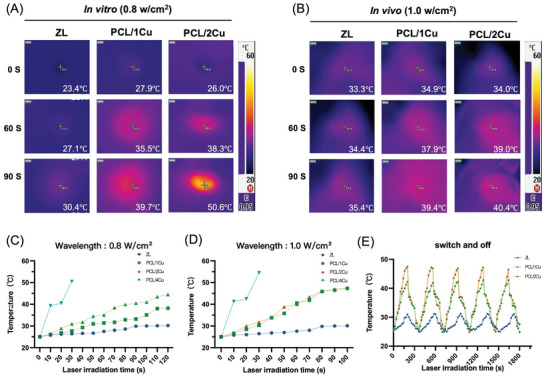
Photothermal properties: Infrared thermographic maps of samples in vitro (A) and in vivo (B) under NIR laser irradiation (808 nm, 1.0 W cm^−2^).; Photothermal temperature curves of ZL and PCL/Cu coatings with different copper concentrations in vitro with 0.8 W cm^−2^ (C) and 1.0 W cm^−2^ (D) power density of NIR laser irradiation (808 nm), respectively; Temperature profiles in vitro during five laser on/off cycles under NIR laser irradiation (808 nm, 1.0 W cm^−2^). n = 3.

**Figure** **3** illustrates the excellent photothermal performance of the coatings both in vitro and subcutaneously in SD rats. The wavelength‐dependent tissue penetration depth of light influences the use of various phototherapies in clinical settings. Because endogenous biomolecules and chromophores like water, blood, and melanin attenuate and refract NIR light less than ultraviolet and visible light, it can penetrate tissue deeper.^[^ ^13b^ ^]^ Rat skin, for instance, has tissue penetration depths of 7.5 ± 0.5 mm at 705 nm, 6.3 ± 0.5 mm at 633 nm, and just 1.0 ± 0.02 mm at 408 nm.^[^ ^28^ ^]^ According to in vivo experiments conducted on SD rats, 808 nm NIR light has the ability to enter the femur's tissue and produce a mild PTT effect, effectively promoting osteogenesis.^[^ ^29^ ^]^ We also explored the effect of NIR laser power density, that is, 0.8 and 1.0 W cm^−^^2^. After 100 s of irradiation, the ZL group maintained a temperature below 30 °C, whereas the PCL/CuS‐coated groups demonstrated heating (Figure 3A,C,D). CuS NPs act as PTA under NIR light irradiation. The heating rate increased with increasing CuS NPs content; the PCL/4Cu group was excluded from subsequent experiments due to rapid heating to more than 50 °C within 30 s. The PCL/1Cu and PCL/2Cu groups underwent a mild photothermal treatment temperature of 40–42 °C after 100 s of irradiation. Based on existing literature, which suggests that 0.8 W cm^−^^2^ is an effective and safe power density for cell studies. For in vivo experiments, we carefully selected a power density of 1 W cm^−^^2^, considering that higher tissue penetration is needed to reach and sustain the target therapeutic temperature of 40–42 °C in SD rat models.^[^ ^30^ ^]^ Upon subcutaneous implantation in SD rats and NIR light irradiation for 90 s, the ZL group exhibited no significant temperature change, maintaining the body temperature of SD rats at ≈35 °C. In contrast, the PCL/1Cu group heated to 39.4 °C and the PCL/2Cu group increased by 5 °C compared with the ZL group (Figure 3B). After 3 min of radiation, the coating was allowed to naturally cool to room temperature. Consistent temperature elevation throughout five laser on/off cycles demonstrated the coating's robust resistance to repeated heating, indicating excellent photothermal stability (Figure 3E).

### 2.4 Cytocompatibility and Osteogenic Efficiency In Vitro

**Figure 4:**
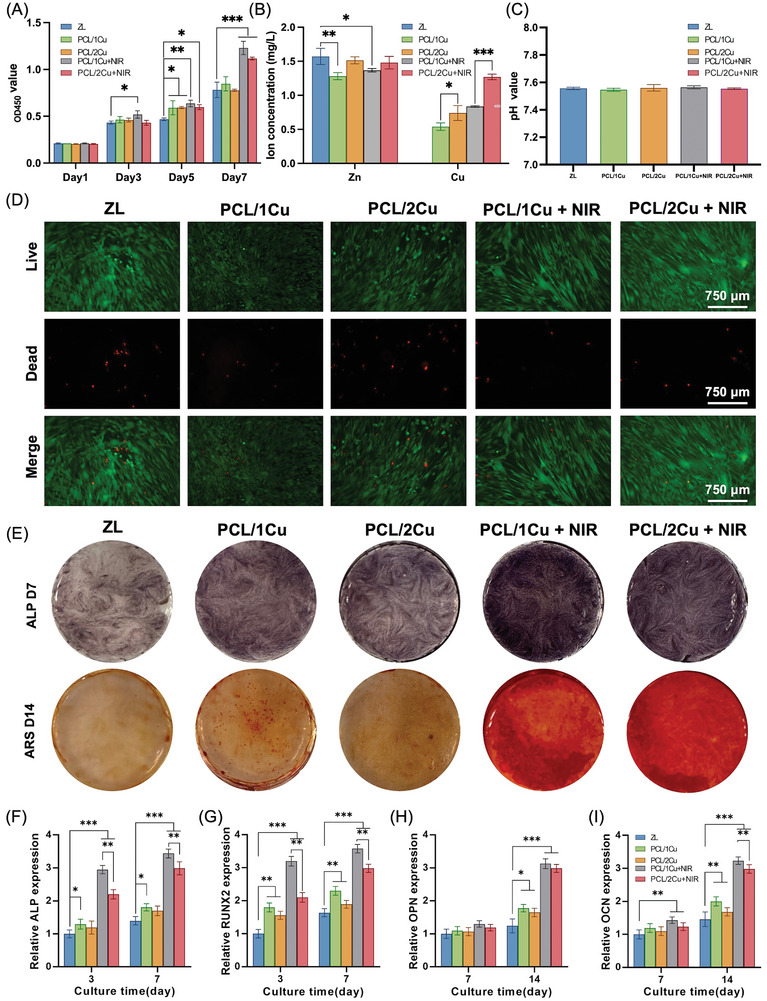
Biocompatibility and Osteogenic efficiency assessment. (A) CCK‐8 assay of hBMSCs cultured with extracts from different groups; (B) Concentration of Zn^2+^ and Cu^2+^ in the extracts from different groups; (C) pH values of extracts from different groups; (D) Live/dead cell staining of hBMSCs. The red cells represent dead cells and the green represent live cells; (E) ALP staining and ARS staining of hBMSCs cultured 7 d and 14 d after osteogenic induction (OI); (F, G, H, I) Expression of genes ALP, RUNX2, OPN, OCN in hBMSCs cultured 3, 7, or 14 d after OI in each group. n = 3 (^*^ *p* < 0.05, ^**^ *p* < 0.01, ^***^ *p* < 0.001, when compared with ZL group).

Using the Cell Counting Kit‐8 (CCK‐8) assay, we also looked into how material extracts from the ZL, PCL/1Cu, PCL/2Cu, PCL/1Cu + NIR, and PCL/2Cu + NIR groups affected the viability of the cells (**Figure** **4**A). When compared to the ZL group, the PCL/1Cu + NIR group demonstrated the best cell compatibility and significantly accelerated cell proliferation (*P* < 0.001). Figure 4B shows the extracts' examination of the concentrations of Zn and Cu ions. While the Zn ion concentration in the extracts of the PCL/2Cu and PCL/2Cu + NIR groups decreased but did not substantially differ from the ZL group, it was significantly lower in the PCL/1Cu and PCL/1cu + NIR groups. The Cu ion concentrations varied among the different experimental groups: 1Cu had the lowest concentration at 0.54 ± 0.05 mg L^−1^, followed by 1Cu+NIR at 0.84 ± 0.01 mg L^−1^, 2Cu at 0.74 ± 0.11 mg L^−1^, and 2Cu+NIR at 1.27 ± 0.04 mg L^−1^. Although there was a modest change in pH between the extracts of the other groups and the ZL group (7.56 ± 0.01), it was not obvious (Figure 4C). Live/dead cell staining revealed that after 24 h of culture, the PCL/1Cu and PCL/1Cu + NIR groups had fewer dead human bone marrow mesenchymal stem cells (hBMSCs) compared with other groups, consistent with the CCK‐8 results (Figure 4D).

Cu is a trace element of human bodies; studies have shown that deficiencies in it impair osteogenesis and cause osteoporotic alterations in the long bones of humans and other animals.^[^ ^17^, ^31^ ^]^ CuS has been reported to exhibit good photothermal conductivity.^[^ ^15a,c^ ^]^ We used CuS to capture light energy and transform it into thermal energy, allowing us to build a PCL/CuS photoresponsive coating that raises the material's surface temperature. Cu^2+^, beyond their photosensitizing role, exhibits osteogenic effects through several mechanisms. Studies have demonstrated that Cu^2+^ could promote osteoblast proliferation and differentiation, leading to increased mineralization and bone formation.^[^ ^17a^ ^]^ Cu^2+^ can also promote angiogenesis, helping create new blood vessels, and is essential for providing nutrients and oxygen to growing bone structures.^[^ ^18b,c^ ^]^ These multifaceted effects underscore the potential of Cu^2+^ in promoting bone growth and regeneration, highlighting their significance in biomaterial applications for bone tissue engineering. However, the release of copper ions indeed brings potential cytotoxic risks. In biomedical applications, the concentration of copper ions must be precisely controlled, as an excess can led to oxidative stress, impair cellular function, and even induce apoptosis. Therefore, the safe concentration range of Cu^2+^ is a crucial consideration in material design.^[^ ^32^ ^]^ In our system, we adjusted the synthesis ratio of PDA to CuS NPs to create the PCL/1Cu, PCL/2Cu, and PCL/4Cu groups. Through a comprehensive evaluation involving a photothermal effect test, Live/Dead staining, CCK‐8 assays, and copper ion concentration measurements in extraction solutions, we ultimately selected the PCL/1Cu group for our in vivo experiments.

Bone growth and development are significantly impacted by Zn, another essential trace metal found in the human body, which makes up 30% of the bone.^[^ ^2^, ^33^ ^]^ At low concentrations, Zn^2+^ promotes bone integration, whereas at high concentrations, it is cytotoxic.^[^ ^19^ ^]^ According to studies, Zn^2+^ with low concentration can promote osteoblast differentiation and proliferation, as well as enhance the deposition and mineralization of bone matrix. Zn also has a role in the activation and control of certain matrix metalloproteinases and bone morphogenetic proteins, which further regulate the development and regeneration of bone tissue. In vivo, Zn^2+^ promotes bone tissue regeneration and repair by enhancing osteoblast activity, increasing the secretion of collagen and bone matrix proteins by osteoblasts, and regulating osteoblast apoptosis and proliferation. These findings indicate that Zn^2+^ could be used in bone tissue engineering and healing, providing an important theoretical basis for further research and clinical applications in medicine.^[^ ^2^, ^34^ ^]^ However, high intracellular Zn levels can induce apoptosis across various tissues by activating pro‐apoptotic molecules, such as p38 and potassium channels, which leads to cell death. Elevated Zn levels can also hinder energy metabolism, further promoting cellular toxicity and apoptosis.^[^ ^35^ ^]^ Besides, excessive Zn^2^⁺ levels also inhibit osteoblast proliferation and differentiation, negatively affecting bone mineralization and repair. These risks emphasize the need for a carefully controlled Zn^2^⁺ release to support safe, sustained osteogenesis without systemic toxicity.^[^ ^2^ ^]^ The selection of the PCL/1Cu and PCL/1Cu + NIR groups for subsequent in vivo experiments was based on its comparatively lower Zn^2+^ and Cu^2+^ concentrations, aiming to minimize potential cytotoxic effects while still maintaining the desired therapeutic efficacy. This decision reflects a balance between therapeutic effectiveness and safety considerations in the context of biomedical applications.

Alkaline phosphatase (ALP) and Alizarin Red S (ARS) staining demonstrated that when exposed to osteogenic induction, the PCL/CuS coatings significantly enhanced osteogenic capability. Simultaneously, the osteogenic effect declined with increasing CuS NPs content. Furthermore, NIR laser irradiation significantly elevated the osteogenic potential of the coatings (Figure 4E). The gene expression levels of osteogenic indicators ALP, runt‐related transcription factor 2 (RUNX2), osteocalcin (OCN), and osteopontin (OPN) in quantitative reverse‐transcription polymerase chain reaction (qRT‐PCR) were found to be consistent with the staining results after 3, 7, and 14 days of culture in osteogenic medium, as shown in Figure 4F–I.

### 2.5 Promoting Osteointegration and Degradation Behavior In Vivo

**Figure 5:**
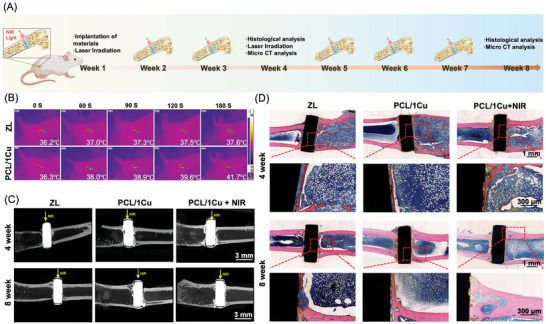
In vivo osseointegration promotion studies. (A) Diagram of the in vivo experiments in the SD rat femur model, where the implantation site was irradiated with NIR laser (808 nm, 1.0 W cm^−2^, heated to 40–42 °C and held for 5 min) weekly, and the SD rats were euthanized at 4 and 8 w after implantation for subsequent analysis; (B) Infrared thermographic maps of implantation site; (C) Micro‐CT images of the femurs with materials of SD rats; (D) Hard tissue sections of the bone‐implant interfaces after various implantation periods. The red rectangles are zoomed in for clarity.

Considering the in vitro osteogenic effects of each group and the potential cytotoxicity associated with excessive Zn^2+^ and Cu^2+^, the in vivo experimental groups were categorized as ZL, PCL/1Cu, and PCL/1Cu + NIR. We established SD rat femoral defect models and implanted the corresponding materials. The tissue temperature at the implantation site was increased to 40–42 °C with the weekly administration of NIR laser irradiation at a wavelength of 808 nm and intensity of 1.0 W cm^−2^, which was maintained for 5 min. Tissue samples were collected at 4‐ and 8‐weeks post‐implantation. **Figure** **5**A illustrates a schematic diagram of the in vivo experiment, and the soft radiograph of Figure S1 (Supporting Information) confirms the consistency of the material implantation position. Figure 5B shows that the materials displayed effective photothermal performance even through the skin. After 180 s of NIR laser irradiation, the ZL group showed no noticeable temperature increase, whereas the PCL/1Cu group reached 41.7 °C. Morphological changes in femoral bones were observed using micro‐computed tomography (Micro‐CT) and histological examination. The Micro‐CT pictures verified superior bone integration in the PCL/1Cu + NIR group compared to the ZL and PCL/1Cu groups. Interestingly, more new bone and better osseointegration were observed near the NIR light application site in the PCL/1Cu + NIR group (Figure 5C).

Histological staining using methylene blue and basic fuchsin was performed to study the tissue response to different groups (Figure 5D). Both the ZL and PCL/1Cu groups had a layer of fibrous connective tissue between the implant and bone tissue four weeks after surgery, indicating inadequate osseointegration. Conversely, in the PCL/1Cu + NIR group, the implant closely contacted the surrounding bone tissue. Additionally, the PCL/1Cu + NIR group exhibited more and thicker new bone deposition around the implant. With prolonged implantation time, all groups showed some degree of bone integration and new bone deposition by week 8. However, the ZL and PCL/1Cu groups still exhibited partial fibrous connective tissue, indicating suboptimal bone integration. In contrast, the PCL/1Cu + NIR group showed no obvious fibrotic layer adjacent to the implant, and more new bone tissue was observed, demonstrating superior promotion of osseointegration and osteogenesis.

**Figure 6:**
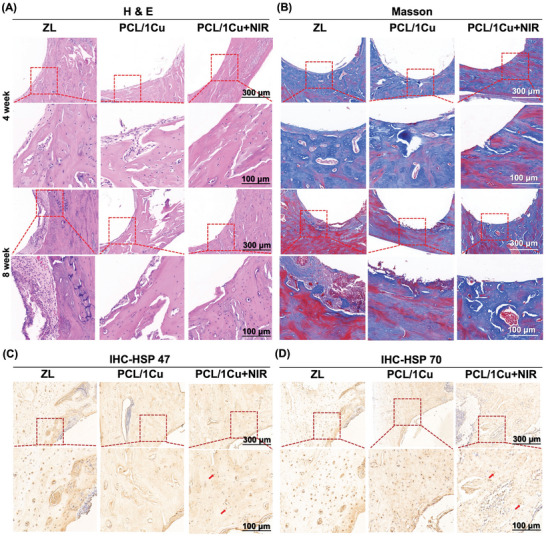
Histological analysis of decalcified samples. (A) H&E and (B) Masson's trichrome staining of ZL, PCL/1Cu, and PCL/1Cu + NIR after 4‐ and 8‐week implantation. (C) HSP 47 and (D) HSP 70 immunohistochemical staining of femur defects at 4 weeks after surgery. (Arrow: Dark brown granules in the cytoplasm and around the nuclei. The red rectangles are magnified.).

Four and eight weeks after implantation, Hematoxylin and Eosin (H&E) staining was carried out to examine the inflammatory response. The ZL and PCL/1Cu groups showed local inflammatory infiltration of macrophages and lymphocytes surrounding the implant at 4 and 8 weeks post‐operatively, whereas fewer inflammation cells were detected in the PCL/1Cu + NIR group (**Figure** **6**A). Additionally, an assessment of the maturity of the newly formed bone surrounding the implants was conducted using Masson‐Trichrome staining. After 4 weeks of implantation, both the ZL and PCL/1Cu groups exhibited more mature bone around the implants, with no apparent, red‐colored new bone tissue. However, some fibrous tissues were still visible. The PCL/1Cu + NIR group showed more new bone formation without visible fibrous tissue, indicating its excellent ability to promote osseointegration and osteogenesis. Interestingly, at 8 weeks post‐implantation, the new bone around the PCL/1Cu + NIR group appeared more mature, with more blue‐colored mature bone tissue. Compared to the PCL/1Cu + NIR group, the ZL, and PCL/1Cu groups showed more juvenile bone and the mature bone tissue was thinner (Figure 6B).

H&E staining after 4 and 8 weeks of implantation showed no significant pathological changes, such as tissue damage or inflammation, in major organs, including the heart, liver, spleen, lung, and kidney (Figure S2, Supporting Information), confirming the biosafety of our implant materials over this period. Additionally, the PCL/1Cu + NIR group demonstrated enhanced osseointegration in the SD rat model. While these results are promising, they are based on an 8‐week in vivo study, and prior studies have shown the biosafety of Zn alloys in longer‐term applications, up to 12 months.^[^ ^36^ ^]^ However, the SD rat model has limitations due to structural differences between rat and human bone, and may not fully predict clinical outcomes in humans. We plan to conduct further studies by extending the implantation period and using larger animal models to better evaluate the material's potential for clinical translation.

Several studies have proposed strategies to accelerate bone tissue regeneration through NIR‐photothermal therapy, particularly the application of mild heat. The mild heat produced by thermotherapy promotes the creation of new bone and increases cortical bone density.^[^ ^13^, ^29^, ^37^ ^]^ Similarly, the expression of ALP in BMSCs and the development of mineralized nodules increases significantly with mild heat application.^[^ ^38^ ^]^ It has been suggested that stem and progenitor cells are susceptible to modulatory aspects of NIR laser treatment, which induces photon uptake in cellular mitochondria and activates a series of signals involving a range of transcription factors.^[^ ^39^ ^]^ It is confirmed that NIR light can stimulate osteogenic differentiation by the upregulation of genes involved in osteogenic differentiation, cell proliferation, and tissue repair.^[^ ^40^ ^]^ Heat shock proteins (HSPs), particularly HSP70 and HSP47, can be upregulated when mild photothermal treatment is administered via NIR ligt.^[^ ^38^, ^41^ ^]^ These two heat shock proteins play crucial roles during the treatment process. The immunohistochemical staining results after 4 weeks of implantation showed more dark brown‐stained particles specific to HSP 47 (Figure 6C) and HSP 70 (Figure 6D) in the PCL/1Cu + NIR group. It is reported that exposure to mild heat induces the upregulation of HSP expression, particularly HSP47 and HSP70, in bone cells and mesenchymal stem cells (MSCs).^[^ ^42^ ^]^ HSP47 is known to facilitate the proper folding and secretion of collagen, an essential component of bone extracellular matrix (ECM), thereby enhancing ECM remodeling and mineralization during bone regeneration.^[^ ^43^ ^]^ On the other hand, HSP70 acts as a molecular chaperone, assisting in the refolding of denatured proteins and protecting cells from stress‐induced damage, which is crucial for maintaining cell viability and function during the regenerative process.^[^ ^44^ ^]^ Furthermore, HSPs have been implicated in modulating intracellular signaling pathways involved in osteogenic differentiation and bone formation. HSP70 has been shown to interact with key regulators of bone morphogenetic protein (BMP) signaling, enhancing BMP‐mediated osteogenic differentiation of MSCs and promoting bone formation.^[^ ^37^, ^41^ ^]^ By facilitating proper protein folding and modulating intracellular signaling, HSP47, and HSP70 contribute to the overall pro‐osteogenic effects of mild photothermal therapy, ultimately promoting bone tissue regeneration.

The biocompatibility of degradation products during SD rat metabolism and excretion was evaluated histologically. At 4 and 8 weeks after implantation, there were no obvious signs of tissue damage, lesions, or inflammation in the heart, liver, spleen, lungs, and kidneys of all groups (Figure S2, Supporting Information). These results confirm the biocompatibility of the materials, without observable functional impairment or organ pathology.

**Figure 7:**
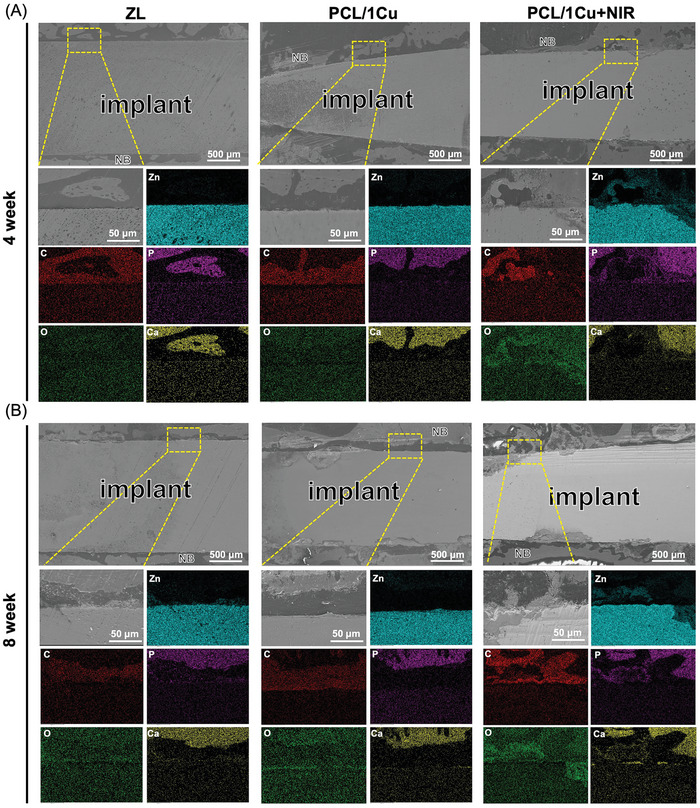
SEM images of the cross‐sections of ZL, PCL/1Cu, and PCL/1Cu + NIR for (A) 4 weeks and (B) 8 weeks. NB: new bone. In addition, the lower panels show corresponding EDS maps of elements of interest for the yellow rectangular areas.

Using SEM and EDS mapping, we examined cross‐sections between implants and bone to investigate alterations in the composition and structure of the degradation layer (**Figure** **7**). Only the PCL/1Cu + NIR group showed evidence of the tight interaction between the implant and the bone tissue. All three groups showed signs of degradation products spreading into the surrounding tissues after 8 weeks of surgery. This trend was similar to that of the 4‐week results, the PCL/1Cu + NIR group indicated superior bone integration with thicker and denser new bone formation.

### 2.6 PCL/1Cu + NIR Regulated Osteogenesis Via the Wnt/β‐Catenin and NF‐kB Signaling Pathways

**Figure 8:**
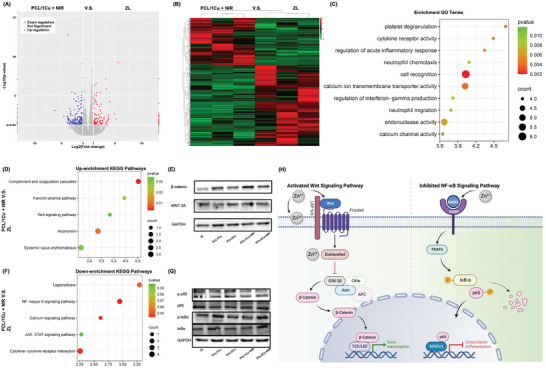
Gene expression profiles between PCL/1Cu + NIR group and ZL group. (A) Volcano map of differentially expressed genes; (B) Heatmap for global gene expression; (C) GO enrichment analysis of the top 10 different genes; (D) KEGG pathway analysis of up‐regulated genes; (F) KEGG pathway analysis of down‐regulated genes; (E, G) Western blot of β‐catenin and WNT‐3A in hBMSCs treated with different methods for 10 days; (H) Schematic diagram of Zn^2+^ action on Wnt/β‐catenin and NF‐κB signaling pathway. n = 3.

RNA sequencing analysis was carried out to identify differentially expressed genes in hBMSCs under different treatment conditions, investigating the molecular mechanisms behind the stimulation of osseointegration by PCL/1Cu + NIR (**Figure** **8**A−F). The findings showed that the levels of gene expression of ZL and PCL/1Cu + NIR groups differed significantly (Figure 8B). Wnt11 and CCL19 gene expression were shown to be upregulated and downregulated in the experimental group, respectively, according to Gene Ontology and Kyoto Encyclopedia of Genes and Genomes (KEGG) pathway studies of these genes (Figure 8C,D,F). Previous research indicates that mesenchymal stem cell osteogenic differentiation is promoted by the Wnt11 gene via the Wnt/β‐catenin signaling pathway.^[^ ^45^ ^]^ Other studies indicated that CCL19 can induce the phosphorylation of NF‐κB, whose primary role in bone is to promote osteoclast formation and inhibit osteoblast differentiation.^[^ ^45^, ^46^ ^]^ Following 7 days of osteogenic induction, Western blot analysis was carried out to examine the protein expression of key molecules within the Wnt/β‐catenin pathway.β‐catenin and WNT‐3A were higher expressed in the PCL/1Cu + NIR group (Figure 8E). To evaluate the expression levels of NF‐κB‐related proteins, we examined the levels of p‐p65 and p‐IκBα, finding that the PCL/1Cu + NIR group inhibited the NF‐κB signaling pathway, consistent with RNA sequencing results (Figure 8G). These findings suggest that the experimental group regulates osteogenic differentiation by activating the Wnt/β‐catenin pathway while inhibiting the NF‐κB signaling pathway (Figure 8H).

## 3 Conclusion

In this study, we developed a novel NIR light‐controlled PCL/Cu‐coated ZL alloy suitable for orthopedic applications that address the issues of degradation. PCL is used to encapsulate the Zn–Li alloy, providing a protective effect, and leading to slow down degradation rate. With NIR light irradiation, light energy is transferred into heat and realized the mild photothermal therapy with CuS NPs. Osseointegration was improved through the combined effect of mild photothermal therapy and Zn^2+^. Rigorous in vitro and in vivo evaluations confirmed the enhanced osteogenic potential and superior osseointegration of the biomaterial. Our results revealed that the NIR light‐controlled PCL/CuS coated ZL alloy have a promising future in orthopedic applications.

## 4 Experimental Section

### PCL/CuS/Zn‐0.1Li Preparation

Ingots of the Zn‐0.1Li (wt%) binary alloy were acquired from the lab of Y. Zheng (Peking University, Beijing, China). Round samples (10 mm diameter × 2 mm length) cut from extruded rods were used for in vitro experiments. Cylindrical samples with a diameter of 2 mm and a length of 6 mm were utilized for in vivo investigations.

For the PCL/CuS coating, at room temperature, 1000 mL of an aqueous solution containing sodium citrate (0.68 mM; Hushi) and CuCl_2_ (1 mM; Hushi) was combined with 1 mL of Na_2_S solution (1 M; Hushi) and agitated. The reaction mixture was agitated for 15 min and heated to 90 °C after 5 min. After being created, citrate‐coated CuS NPs were kept at 4 °C. PDA (2 mg mL^−1^; Aladdin, China) was dissolved in 10 mM Tris‐HCl (pH 8.5; Aladdin) and the Cit‐CuS NPs were mixed with the PDA solution at a volume ratio of 1:1, 2:1, or 4:1 (denoted as 1Cu, 2Cu, or 4Cu, respectively). The ground ZL alloy was submerged in the mixture and left for 12 h at room temperature with light protection. After that, the coated surface was dried in N_2_ gas, rinsed with deionized water, and stored. To create a homogenous polycaprolactone methacryloyl/2,4,6‐trimethylbenzoyldiphenylphosphinate (PCLMA/TPO) solution, TPO (Engineering For Life) and PCLMA (PCLMA‐3200; Engineering For Life, China) were combined at a weight ratio of 0.5:100 after being heated to 40°C. For further testing, an even 50 mg cm^−2^ thin layer of the combination was applied to the CuS/ZL surface, and it was photocured with a 405 nm light source.

### Surface Characterization and Composition Analysis

The elemental composition and surface morphology of the samples were analyzed using an EDS‐equipped SEM (Gemini 300; Zeiss, Germany). The average size of the NPs was measured by DLS (ZetaSizer Nano ZS90, Malvern Instruments, Britain). Utilizing Cu‐Kα radiation at a scanning rate of 2° min^−1^ within the 2θ range of 5°–90° at 40 kV and 40 mA, surface phase investigation was performed using XRD (SmartLab SE; Rigaku, Japan). The surface chemical composition was determined by XPS (K‐Alpha; Thermo Scientific, USA). The samples' 3D surface topography was examined using Dimension Icon atomic force microscopy (Bruker, Germany). Using a water‐air contact angle system (model OCA20; DataPhysics Instruments, Germany), the hydrophilicity of the surface was assessed. After 30 s, the water‐air contact angle was measured on the sample surface with a 1‐µL water drop applied.

### Immersion Tests

The samples were submerged in SBF at a ratio of 20 mL cm^−2^ at 37 °C for a maximum of 28 d. The SBF composition closely mimics human plasma, containing NaCl (142.0 mMm), KCl (5.0 mMm), CaCl₂ (2.5 mMm), MgCl₂·6H₂O (1.5 mMm), NaHCO₃ (4.2 mMm), K₂HPO₄·3H₂O (1.0 mMm), and Na₂SO₄ (0.5 mMm), with the pH adjusted to 7.4 using 50 m m HCl. The pH and Zn^2+^ concentration in the extracts were measured using a pH meter (SevenCompact; Mettler Toledo, USA) and inductively coupled plasma emission spectrometry (ICP; model iCAP6300; Thermo Fisher Scientific, USA). After the samples were submerged, they were collected and cleaned with a CrO_3_ solution, and air dried. SEM and EDS were then used to investigate changes in surface morphology and chemical composition. The corrosion rates of the samples were determined by measuring weight loss with the equation: $C=\frac{\Delta m}{\rho \cdot A\cdot t}$, where *C* is the corrosion rate in mm/year, Δ*m* is the weight loss, ρ is the material density, *A* is the initial implant surface area, and *t* is the implantation time. For each group, the average was calculated from at least three measurements.

### Photothermal Property Tests

Using an infrared thermal imaging camera, infrared thermograms and temperature changes of the materials were recorded. (model 875‐1i; Testo, Germany). The 808 nm NIR light was used to irradiate samples of ZL, PCL/1Cu, PCL/2Cu, and PCL/4Cu at power densities of 0.8 and 1.0 W cm^−2^. After 3 min‐irradiation, the NIR laser (808 nm, 1.0 W cm^−2^) was turned off to allow the sample to cool to room temperature during 3 min. 5 cycles of this switching pattern were performed to assess the PCL/Cu coating's photothermal stability. Subsequently, the material was implanted under the skin of SD rats and exposed to 1.0 W cm^−2^ of irradiation to observe the photothermal effect through the skin.

### Biocompatibility Assessment In Vitro

The hBMSCs (ScienCell, USA) were grown in α‐minimum essential medium (α‐MEM; Gibco, USA) was cultured. 10% (v/v) fetal bovine serum (FBS) and 1% (v/v) antibiotics made up the proliferation medium (PM). Osteogenic medium (OM) was employed with 10% (v/v) fetal bovine serum, 1% (v/v) antibiotics, 10 nM dexamethasone, 200 µM ascorbic acid, and 10 mM β‐glycerophosphate to induce osteogenic differentiation. The sample was subjected to at least 1 h of UV radiation on each side before usage. The materials were submerged in α‐MEM cell culture medium for 24 h, with an extraction ratio of 1.25 mL cm^−2^, following conventional cell culture procedures, to yield material extracts. Subsequent experiments were conducted using a 10% extraction solution. ICP was used to measure the ion concentrations in the mixed extract, which was made from a minimum of three samples.

A 100 µL cell solution was seeded at a density of 3 × 10^4^ cells mL^−1^ into 96‐well cell culture plates for cell viability tests. Once every 3 days, the material extracts were added to the cell culture medium after it had been incubated for 24 h, and the cells were exposed to an NIR laser (808 nm, 0.8 W cm^−2^, 5 min). The incubation period for the cells was 1, 3, 5, and 7 days. The material extracts were replaced at the conclusion of each time point with cell culture media containing 10% CCK‐8 (Dojindo, Japan), and the cells were incubated for 1 h at 37 °C. A microplate reader (Model 680; Bio‐Rad, USA) was used to measure each group's spectrophotometric absorbance at 450 nm. For cell viability, at least five duplicates were used.

Following a day of incubation with the material extracts, the cells were rinsed three times with phosphate‐buffered saline (PBS) and then incubated for 30 min at room temperature with 2 µM calcein‐AM and 8 µM propidium iodide (Animal Cell Live/Dead Viability/Cytotoxicity Assay Kit; KeyGen Biotechnology, China). After that, the cells were examined using fluorescent microscopy (Eclipse Ti‐S; Nikon, Japan) after being rinsed three times with PBS. Three observers examined each sample.

### Osteogenic Efficiency In Vitro—ALP and ARS Staining

Cells were seeded at a density of 2 × 10^4^ cells per well in 12‐well plates and exposed to material extracts for ALP and ARS staining tests. NIR laser irradiation (808 nm, 0.8 W cm^−2^, 5 min) was applied to the PCL/1Cu + NIR, PCL/2Cu + NIR groups once every 3 days post‐osteogenic induction. For ALP staining, the nitro blue tetrazolium (NBT)/5‐bromo‐4‐chloro‐3‐indolyl phosphate (BCIP) staining kit (Beyotime, China) was employed on samples at the 7‐day mark. Then, at 14 days of osteogenic induction, specimens were stained with a 2% Alizarin Red buffer (Aladdin).

### qRT‐PCR

Cells were cultured in six‐well plates at a density of 5 × 10^4^ cells per well. Total cellular RNA was extracted using TRIzol reagent (Invitrogen, USA) from cells cultivated for 3, 7, and 14 days. RT‐PCR was carried out using the ABI Prism 7500 RT‐PCR system (Applied Biosystems, USA) with SYBR Green Master Mix, using GAPDH as the reference gene. The primer sequences for human GAPDH, ALP, OPN, OCN, and RUNX2 are listed in **Table** **1**.

**Table 1:** Primer sequences for RT‐PCR.

Target gene	Forward Primer [5‘‐3’]	Reverse Primer [3‘‐5’]
GAPDH ^a)^	AAGGTCGGAGTCAACGGATTTG	TCCTGGAAGATGGTGATGGGAT
ALP	ATGGGATGGGTGTCTCCACA	CCACGAAGGGGAACTTGTC
OPN ^b)^	ACCCTTCCAAGTAAGTCC	TGTCCTCGTCTGTAGCAT
OCN ^c)^	AGCCACCGAGACACCATGAGA	GGCTGCACCTTTGCTGGACT
RUNX2 ^d)^	ACTACCAGCCACCGAGACCA	ACTGCTTGCAGCCTTAAATGACTCT

^a)^ GAPDH: glyceraldehyde‐3‐phosphate dehydrogenase

^b)^ OPN: osteopontin

^c)^ OCN: osteocalcin

^d)^ RUNX2: runt‐related transcription factor 2

### RNA Sequencing

In this study, hBMSCs were cultured for 7 days in the extracts from the ZL group, PCL/1Cu group, PCL/2Cu group, PCL/1Cu + NIR group, and PCL/2Cu + NIR group. Total RNA was isolated using TRIzol reagent (from Invitrogen) according to the manufacturer's instructions. Each sample was sequenced three times. RNA sequencing was conducted by Shanghai Biotechnology Corporation (Shanghai, China). Reads containing adapters or ploy‐Nb sequences, as well as low‐quality reads, were removed to ensure clean and high‐quality data for subsequent analysis. Differential gene expression analysis was performed using DEGSeq2 software (version 1.20.0), with a significance threshold set at a corrected p‐value of 0.05 and an absolute fold change of 2. The distribution of differentially expressed genes in KEGG pathways was analyzed using the clusterProfiler R package (version 3.8.1) to determine important biological mechanisms.

### Western Blot Analysis

Cells were seeded at a density of  ×10^5^ per 6‐cm dish. To detect nuclear proteins, cells were lysed using a lysis buffer containing 2% protease and phosphatase inhibitors, specifically to isolate nuclear protein fractions. Protein concentrations were measured with the BCA Protein Assay Kit (Thermo Scientific). Equal amounts of nuclear protein were separated by 10% SDS‐PAGE and transferred to a PVDF membrane. Membranes containing nuclear extracts from hBMSCs were incubated overnight at 4 °C with primary antibodies specific to nuclear markers β‐catenin, WNT‐3A, p65, phospho‐p65 (p‐p65), IκBα, phosphorylated IκBα (p‐IκBα), and GAPDH (Proteintech, China). After applying peroxidase‐conjugated secondary antibodies at room temperature, immunoreactive protein bands were visualized using an enhanced chemiluminescence (ECL) kit (CWBIO, China).

### Osteogenic Efficiency In Vivo

All animal experimental procedures and experiments were approved by the Ethics Committee of the Faculty of Medicine, Peking University (LA2021006). Animal experiments were performed according to the protocol established by the Experimental Animal Ethics Division. 24 8‐week‐old male SD rats were randomly divided into three groups: ZL, PCL/1Cu, and PCL/1Cu + NIR. NIR laser irradiation (808 nm, 1.0 W cm^−2^) applied to the PCL/1Cu + NIR group once per week raised the temperature around the tissue to 40–42 °C, which was maintained for 5 min. Prior to surgery, each rat was given 50 mg kg^−1^ of sodium pentobarbital to induce anesthesia. Each sample was inserted into a drilled bone tunnel on the femoral condyles, measuring 2 mm in diameter by 6 mm in height. 48 samples in all were implanted. Following surgery, each rat was housed in an animal facility with regulated environmental conditions. Four and eight weeks after implantation, the rats were put to death. For 24 h at room temperature, femurs, hearts, livers, spleens, lungs, and kidneys were fixed in 10% neutral formalin buffer. Twelve rat femurs in total were gathered at each time point.

After being rinsed in water for 24 h, half of the femur samples were gradually dehydrated in 70% to 100% ethanol and then embedded in polymethylmethacrylate (PMMA). Following embedding, the samples were sectioned using an EXAKT 300CP saw microtome (Leica, Germany) into 200‐µm‐thick slices. Prior to histological analysis, these sections were polished and ground to a thickness of 20 µm. They were then stained with methylene‐blue/acid fuchsin.

After 6 weeks of decalcification at ambient temperature in a 10% ethylenediaminetetraacetic acid solution (pH 7.4), the remaining femur samples underwent dehydration, transparency, and paraffin embedding. Using a microtome, slices with a thickness of 5 µm were produced. Similarly, paraffin was used to embed fixed organ samples (hearts, livers, spleens, lungs, and kidneys) to create sections that were 2 to 3 µm thick. For histological examinations, femur samples underwent Masson, H&E, and immunohistochemistry (IHC) staining; organ samples underwent H&E staining. ZEN‐BIO provided the HSP47/70 primary antibodies utilized in IHC staining (Zen‐Bioscience, China). An optical microscope (BX51; Olympus, Japan) was used to examine the captured images.

### Statistical Analysis

The statistical software SPSS 21.0 (SPSS, USA) was used to conduct the analyses. After comparing means using one‐way analysis of variance (ANOVA), significant differences were determined using Tukey's post hoc tests, with a threshold of *P* < 0.05.

## Conflict of Interest

The authors declare no conflict of interest.

## Supporting information



Supporting Information

## Data Availability

The data that support the findings of this study are available from the corresponding author upon reasonable request.
