# Supplementary material for: Photothermal Coating on Zinc Alloy for Controlled Biodegradation and Improved Osseointegration
Source: Adv Sci (Weinh). 2025 Jan 14;12(9):2409051. doi: 10.1002/advs.202409051 (PMC11884568; doi:10.1002/advs.202409051)
Supplement: Supplementary file 1 — Supporting Information [file ADVS-12-2409051-s001.docx]

**Supplementary Information**

**
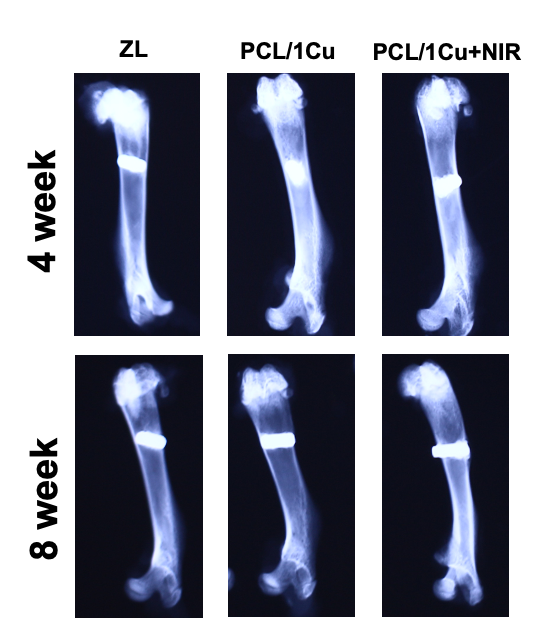
**

Figure S1. Soft X-ray images of femurs from different groups after 4- and 8-week implantation.


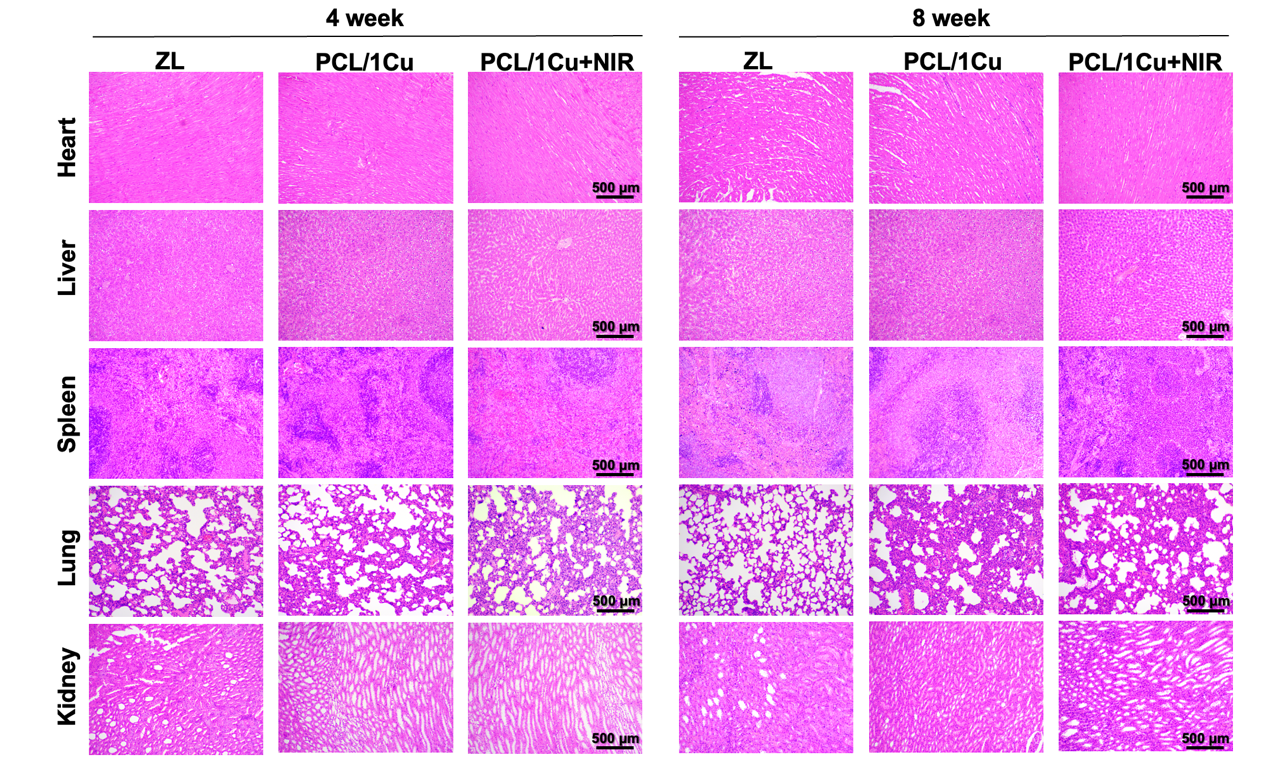


Figure S2. Histological images of diverse organs after 4- and 8- week of implantation of ZL, PCL/1Cu and PCL/1Cu + NIR groups.
